# Event Timing in Associative Learning: From Biochemical Reaction Dynamics to Behavioural Observations

**DOI:** 10.1371/journal.pone.0032885

**Published:** 2012-03-30

**Authors:** Ayse Yarali, Johannes Nehrkorn, Hiromu Tanimoto, Andreas V. M. Herz

**Affiliations:** 1 Max Planck Institute of Neurobiology, Martinsried, Germany; 2 Ludwig-Maximilians-Universität München, Department Biology II, Division of Neurobiology, Martinsried, Germany; 3 Bernstein Center for Computational Neuroscience, Munich, Germany; Freie Universitaet Berlin, Germany

## Abstract

Associative learning relies on event timing. Fruit flies for example, once trained with an odour that precedes electric shock, subsequently avoid this odour (punishment learning); if, on the other hand the odour follows the shock during training, it is approached later on (relief learning). During training, an odour-induced Ca^++^ signal and a shock-induced dopaminergic signal converge in the Kenyon cells, synergistically activating a Ca^++^-calmodulin-sensitive adenylate cyclase, which likely leads to the synaptic plasticity underlying the conditioned avoidance of the odour. In *Aplysia*, the effect of serotonin on the corresponding adenylate cyclase is bi-directionally modulated by Ca^++^, depending on the relative timing of the two inputs. Using a computational approach, we quantitatively explore this biochemical property of the adenylate cyclase and show that it can generate the effect of event timing on associative learning. We overcome the shortage of behavioural data in *Aplysia* and biochemical data in *Drosophila* by combining findings from both systems.

## Introduction

**Figure 1:**
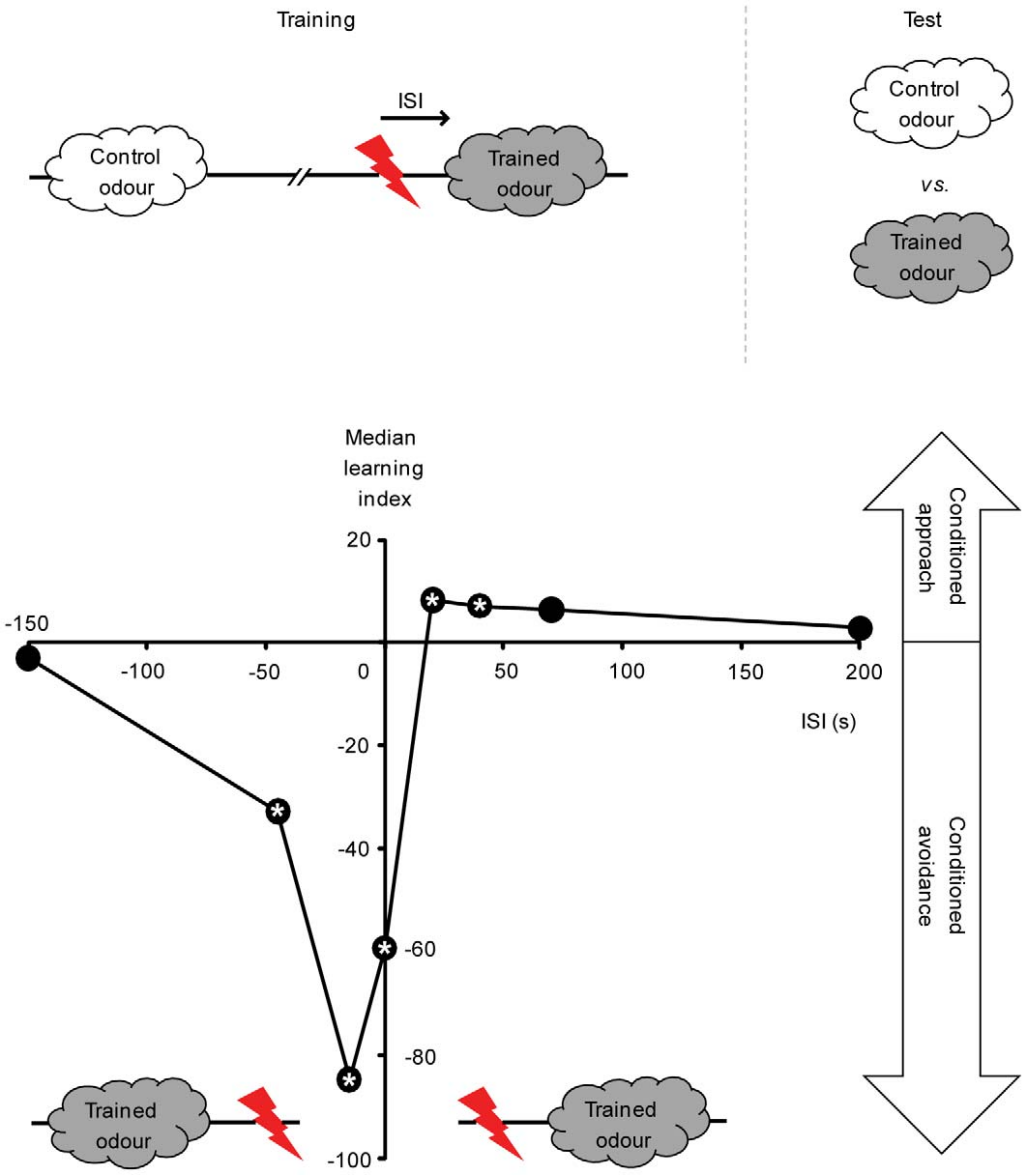
Event timing affects associative learning. Fruit flies are trained such that a control odour is presented alone, whereas a trained odour is paired with pulses of electric shock as reinforcement. Across groups, the inter-stimulus interval (ISI) between the onsets of the trained odour and shock is varied. Here, ISI is defined such that for negative ISI values, the trained odour precedes shock; positive ISI values mean that the trained odour follows shock. For each ISI, two fly subgroups are trained with switched roles for two odours (not shown). During the test, each subgroup is given the choice between the two odours; the difference between their preferences is taken as the learning index. Positive learning indices indicate conditioned approach to the trained odour, negative values reflect conditioned avoidance. Very long training ISIs support no significant conditioned behaviour. If the odour shortly precedes or overlaps with shock during training (ISI = −45 s, −15 s or 0 s), it is strongly avoided in the test (punishment learning). If the odour closely follows the shock-offset during training (ISI = 20 s or 40 s), flies approach it in the test (relief learning). *: *P*<0.05/8 while comparing to zero in a sign test. Sample sizes are N = 8, 24, 34, 47, 24, 35, 12 and 12. Data from [15], with permission from Informa healthcare.

Predicting future events is a key to survival. For example, if a sensory stimulus typically precedes an aversive event, this relationship will be learned to trigger anticipatory behaviour, such as avoidance [1]. On the other hand, a stimulus that occurs after an aversive event has subsided will be learned as a predictor for relief [2], [3] or safety [4], [5] and will induce approach. Event timing, therefore, determines which of the two opposite learned behaviours is established, as shown in various species including man [6]–[12]. *Drosophila* olfactory associative learning is well-suited for studying this phenomenon (Fig. 1) [11], [13]–[17]: Flies learn to avoid an odour that precedes electric shock during training (i.e. punishment learning); whereas an odour that follows the shock is subsequently approached (i.e. relief learning).

In an attempt to explain punishment and relief learning in fruit flies, Drew and Abbott [18] propose a model circuit where the odour activates a large number of pre-synaptic neurons; while the shock impinges upon a common post-synaptic neuron that mediates the conditioned avoidance. For both types of neuron, the authors assume high firing rates that decay over several seconds upon the termination of the respective stimuli. Within this model circuit, a spike-timing-dependent plasticity (STDP) rule operating at the millisecond-scale can account for the effect of relative odour-shock timing on the conditioned behaviour, which occurs at the scale of several seconds. While demonstrating that slowly decaying spiking activity can enable STDP to function over long intervals, this model does not capture fruit fly olfactory learning, as the corresponding empirically measured odour responses in the Kenyon cells are sparse and short-lasting [19]–[21], violating the model's key assumption.

**Figure 2:**
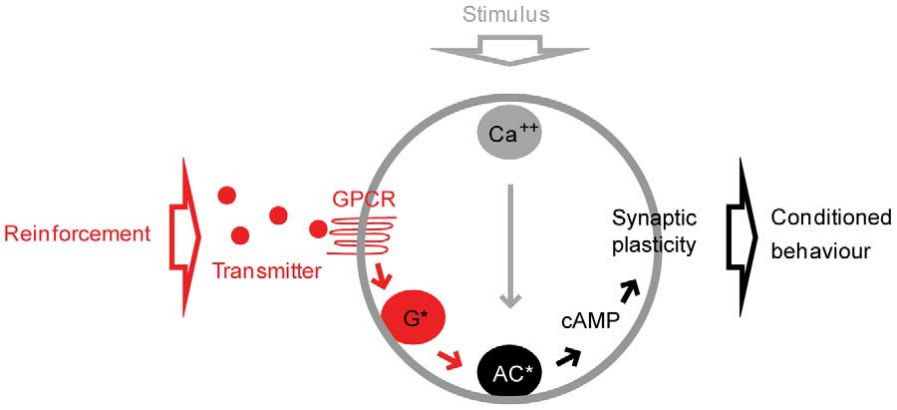
Adenylate cyclase as a molecular coincidence detector. In a variety of associative learning systems, a potential coincidence between the trained stimulus and the reinforcement is detected at the pre-synapse by a particular kind of adenylate cyclase. The stimulus acts on the respective neurons, raising the intracellular Ca^++^ concentration. The reinforcement induces the release of a transmitter that binds to its respective G protein coupled receptors (GPCR) on the very same neurons and activates the G protein (G*). If stimulus and reinforcement are appropriately timed, the two types of input act synergistically on the adenylate cyclase (AC*), triggering cAMP signalling, and thus lead to the strengthening of the output from these neurons to the respective conditioned behaviour pathway.

Here, we propose an alternative model motivated by cellular and biochemical data. In the *Drosophila* brain, individual odours activate small, specific groups of Kenyon cells increasing their intracellular Ca^++^ concentration [22]–[24]; whereas shock induces a dopaminergic reinforcement signal, which is also delivered to the Kenyon cells [25]–[29]. These two inputs likely converge on the Ca^++^-calmodulin-sensitive adenylate cyclase, *rutabaga*; this process seems necessary and sufficient in the Kenyon cells for olfactory learning [30]–[33]. Thus, during punishment training, this adenylate cyclase is synergistically activated in the specific trained odour-responding Kenyon cells [34], [35]; the resulting cAMP signalling then likely strengthens the output from these cells to the conditioned avoidance circuit (Fig. 2) [36]. Those Kenyon cells that respond to a control odour that is presented sufficiently before or after the shock also receive both inputs, but separated in time; consequently, less cAMP is produced [34] and the output of these Kenyon cells is strengthened less, if at all. Then, at test, flies are typically given the choice between the trained odour, which, due to the strengthened output of the respective Kenyon cells, can trigger conditioned avoidance, and the control odour, which does not trigger conditioned avoidance, as the output of the corresponding Kenyon cells has remained weak. To summarize, with respect to punishment learning, a particular, Ca^++^-calmodulin-sensitive adenylate cyclase seems to be the critical detector of the odour-shock convergence.

The biochemical properties of the corresponding Ca^++^-calmodulin-sensitive adenylate cyclase in *Aplysia* (AC-AplA, [37]) have been analyzed in detail. During gill withdrawal reflex conditioning, a Ca^++^ influx due to siphon-touch and a tail-shock-induced serotonergic signal converge on this adenylate cyclase (Fig. 2) [38]–[40], which is sensitive to the relative timing of the two inputs [41]–[43] (see Results for details). We test whether this biochemical phenomenon observed in *Aplysia* (and in rats [44]) can serve as a mechanism for the effect of event timing on associative learning as found in *Drosophila*. A computational approach allows us to overcome the shortage of behavioural data in *Aplysia* and biochemical data in *Drosophila* by combining findings from both systems.

## Results

In an *Aplysia in vitro* neural membrane preparation [41]–[43], a transient serotonin input activates the adenylate cyclase; upon cessation of serotonin, the adenylate cyclase activity returns to the base-line. This effect of serotonin is modified by Ca^++^. If Ca^++^ precedes serotonin by a short time, the adenylate cyclase is activated more rapidly so that the cAMP production exceeds the serotonin-only situation. If, however, Ca^++^ closely follows serotonin, the adenylate cyclase is deactivated faster, resulting in a cAMP production below the serotonin-only case. We implement this property of the adenylate cyclase in two alternative models [45], [46]. This makes it possible to quantitatively explore whether and how far this biochemical phenomenon can explain the effect of event timing on learning; to this end, we simulate a key *Drosophila* experiment (Fig. 1). In addition, we test *in silico* for the effects of parameter changes and relate our results to behavioural findings in *Drosophila*.

### Stimulation of the adenylate cyclase by the transmitter

**Figure 3:**
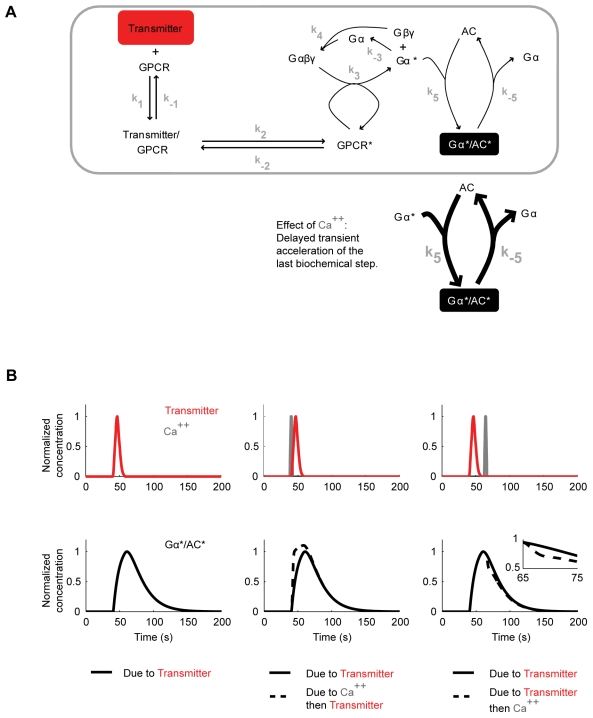
Regulation of the adenylate cyclase by the transmitter and Ca^++^. A. Adapting the model of Rospars et al. [46], the transmitter reversibly binds to its respective G protein coupled receptor (GPCR) to form a complex, resulting in reversible receptor activation (GPCR*). GPRC* catalyzes the dissociation of the trimeric G protein (Gαβγ) into an activated α-subunit (Gα*) and the β- and γ-subunits (Gβγ). Gα* spontaneously deactivates (Gα) and reassembles with Gβγ, or it reversibly interacts with the adenylate cyclase (AC) to form an enzymatically active complex (Gα*/AC*), which serves as the output. Following data from *Aplysia* [41]–[43], Ca^++^ in turn transiently increases the rate constants for both the formation and the dissociation of the Gα*/AC* complex (represented by the thickened arrows). The k_subscript_ denote the rate constants of the respective reactions. B. When this model is stimulated with a transmitter input alone the Gα*/AC* concentration rises to a peak of ∼0.42 molecules/µm^2^ in ∼20 s after stimulus onset, and decays back to zero within the next ∼100 s (left). If a Ca^++^ input immediately precedes the transmitter, the build-up of the Gα*/AC* concentration is transiently accelerated (middle). If on the other hand the Ca^++^ input follows the transmitter, the decay of the Gα*/AC* concentration is transiently accelerated (right). For graphical reasons, normalized concentrations are calculated by dividing with the peak Gα*/AC* concentration given transmitter input alone. The transmitter concentration reaches a peak of ∼6.7·10^4^ molecules/µm^2^ in ∼7 s and decays back to zero within ∼18 s; the Ca^++^ concentration starts rising ∼4.5 s after the onset, reaches a peak value of 5.6·10^−4^ moles/L at ∼6 s and decays back to zero within ∼8.5 s after the onset. Also these inputs are plotted as normalized concentrations.

We use the model by Rospars et al. [46] as a general framework to describe post-receptor G protein signalling. Adapting this model to our case (Fig. 3A), the shock-induced transmitter (Tr) binds to the G protein coupled receptor (GPCR) to form a complex (Tr/GPCR), resulting in receptor activation (GPCR*). GPCR* then dissociates the trimeric G protein (Gαβγ) into an activated α-subunit (Gα*) and the βγ-subunits (Gβγ). Gα* either spontaneously deactivates (Gα) to reassemble with Gβγ, or it interacts with the adenylate cyclase (AC) to form an enzymatically active complex (Gα*/AC*), which is prone to dissociation into inactive AC and Gα. The concentration of the Gα*/AC* complex, i.e. the activated adenylate cyclase, serves as the output variable of the system.

We stimulate this model with a transient transmitter input (Fig. 3B, left; see the Materials and Methods for details), which mimics the *in vitro* experiments in *Aplysia* (Fig. 1A of [42]). When the reaction rate constants k_5_ and k_-5_ are appropriately adjusted (for a detailed sensitivity-analysis, see Fig. 5A), the concentration of Gα*/AC* first rises to a peak within ∼20 s and then decays back to zero within the next ∼100 s (Fig. 3B, left), closely matching the corresponding *Aplysia* data (Fig. 4A of [42]); the deactivation of the adenylate cyclase in the model is slightly slower than the experimental observations.

**Figure 4:**
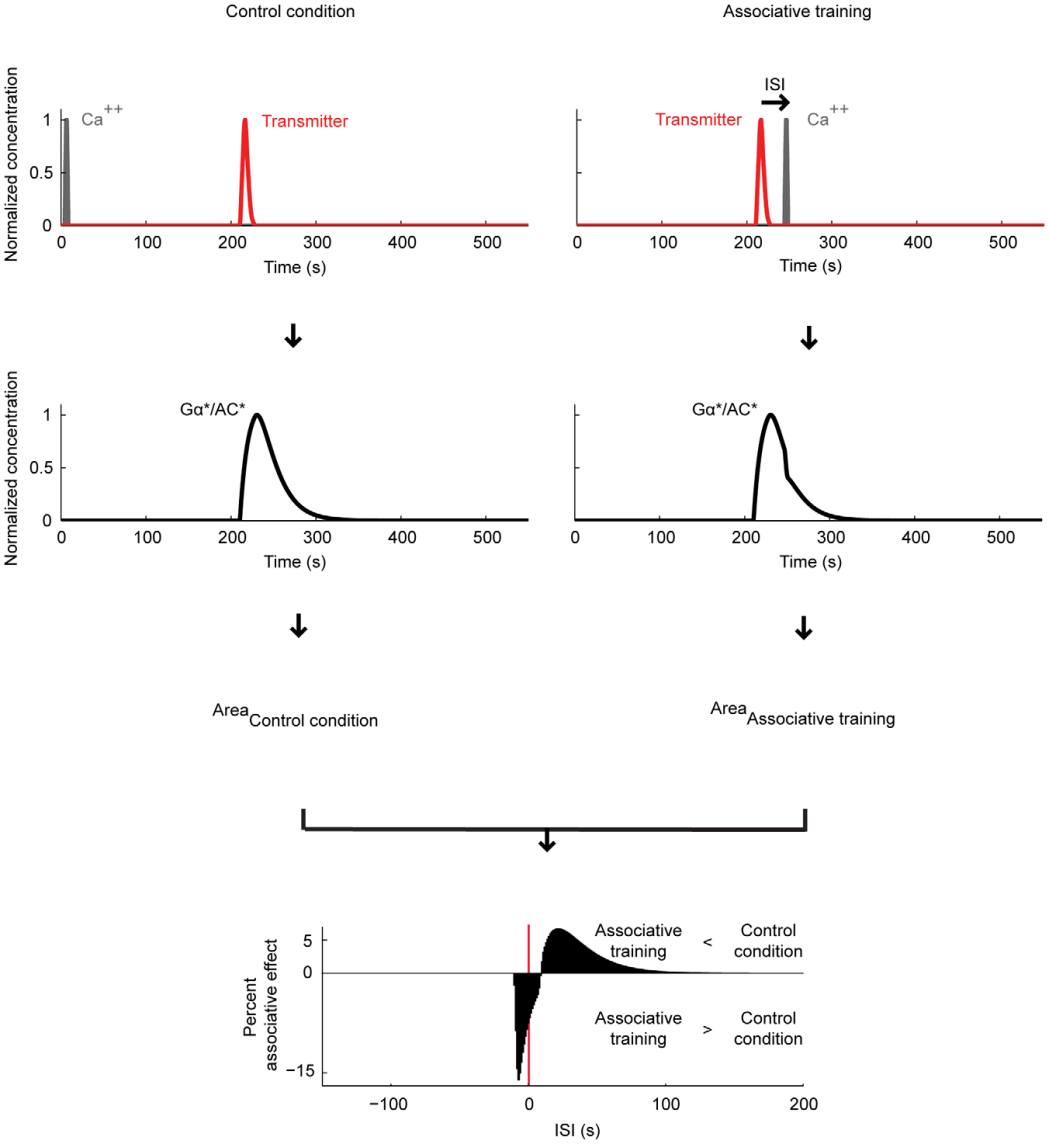
Relative timing of the transmitter and Ca^++^ affects the adenylate cyclase. We stimulate the model with transmitter and Ca^++^ (see Fig. 3B for the details). In the ‘control condition’ (left), Ca^++^ precedes the transmitter by an onset-to-onset interval of 210 s. In ‘associative training’ (right), the two inputs follow each other with an inter-stimulus interval (ISI), which is varied across experiments. Negative ISIs indicate training with first Ca^++^ and then the transmitter; positive ISIs mean the opposite sequence of inputs. For either condition, we take the area under the respective Gα*/AC* concentration curve as a measure of cAMP production. For each ISI, we calculate an ‘associative effect’, by subtracting the amount of cAMP produced during the respective associative training from that in the control condition. We then express the associative effect as percent of the area under the Gα*/AC* concentration curve in the control condition. These percent associative effects are plotted against the ISIs. For very large ISIs, we find no associative effect. If the Ca^++^ is closely paired with the transmitter, we find negative associative effects; the strongest negative associative effect (−15.5%) is obtained when using ISI ∼−3 s. If on the other hand Ca^++^ follows the offset of the transmitter during training, we find positive associative effects; the largest positive associative effect (6.3%) is obtained for ISI ∼26 s. Thus, depending on the relative timing of Ca^++^ and transmitter during training, opposing associative effects come about, closely matching the behavioural situation in Fig. 1.

**Figure 5:**
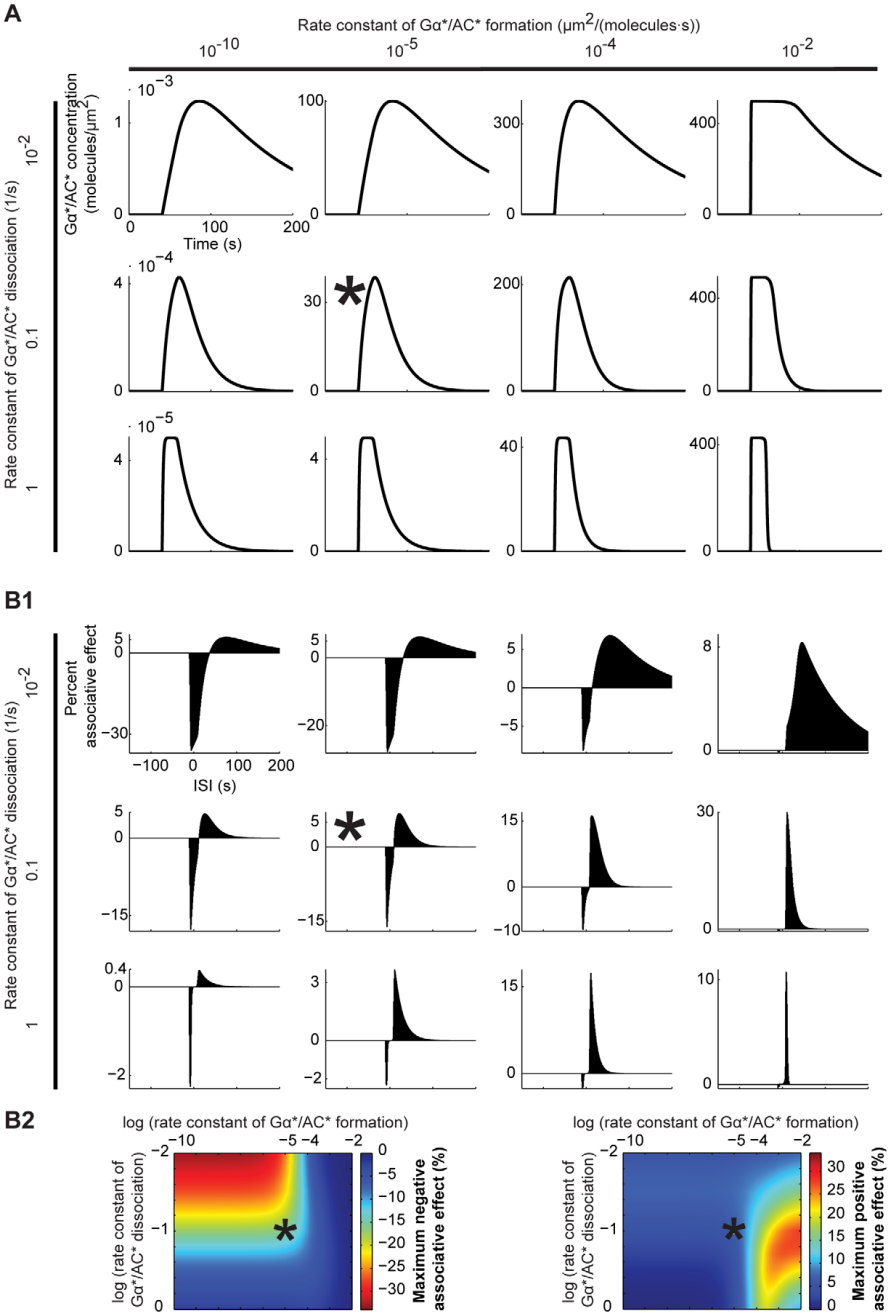
Influence of the rate constants for Gα*/AC* formation and dissociation. A. Time course of the Gα*/AC* concentration, following a stimulation of the model with transmitter (see Fig. 3B for the details). B1. ISI-dependent associative effects, as explained in Fig. 4. B2. Color-coded representation of the size of the peak negative (left) and positive (right) associative effects. In (A), (B1) and (B2), we systematically change the rate constants for Gα*/AC* formation and dissociation (k_5_ and k_-5_ in Fig. 3A). Using the default values of both rate constants, we obtain associative effects fitting the behavioural situation in Fig. 1 (B1, B2: marked with asterices). Notably, this fit is stable over more than five orders of magnitude of the formation rate constant, but is more sensitive to changes in the dissociation rate constant (B1, B2). The size (B2) and ISI-dependency (B1) of the associative effects are dictated by the dynamics of adenylate cyclase activation/deactivation (A). Particularly, the negative associative effect depends on the rising phase of the Gα*/AC* concentration: When either the formation or the dissociation rate constants are increased beyond their default values, the rising of the Gα*/AC* concentration becomes too fast to be further improved by Ca^++^; the negative associative effect is thus attenuated. Also, in this case, the short rising phase of Gα*/AC* concentration limits the window of ISI values appropriate for the negative associative effect. In turn, decreasing both rate constants below their default values slows down the rise of Gα*/AC* concentration, leaving more space for improvement by Ca^++^, thus boosting and -due to the longer rising phase- ‘widening’ the negative associative effect. As for the positive associative effect, the falling phase of the Gα*/AC* concentration matters: When both rate constants are moderately increased beyond their default values, the fall of Gα*/AC* concentration gets faster, that is, the dissociation of Gα*/AC* better dominates over its formation, boosting the positive associative effect. Critically, when the rate constants are increased too much, the drop of Gα*/AC* concentration is accelerated to its limit; thus, both the size and the ‘width’ of the positive associative effect suffer. To summarize, the negative associative effect is favoured by small values of both rate constants, whereas the positive associative effect needs moderately high values of these. Consequently, the overall effect size cannot be improved much beyond the default case, without compromising the relative sizes of the two associative effects with respect to each other and thus the fit to the behavioural situation.

### Effect of Ca^++^

As discussed above, in *Aplysia*, a brief serotonin input results in cAMP production; Ca^++^ in turn bi-directionally modulates the amount of this cAMP production, depending on its timing relative to serotonin [41]–[43]. Critically, at the steady state, Ca^++^ and serotonin have no synergistic effect on cAMP production [41]–[43]. In these biochemical experiments, Ca^++^, bound to calmodulin, seems to interact with the adenlylate cyclase [43] and the Ca^++^-effect on adenylate cyclase is delayed by 2–3 s relative to the effect of serotonin [42], [43]. As a simple way to account for all these findings in our model, we allow Ca^++^ to transiently increase the rate constants for both the formation and the dissociation of the Gα*/AC* complex (k_5_ and k_-5_) with a delay of 2.5 s (Fig. 3A; see the Materials and Methods for details). For simplicity we exclude from our model the biochemical step(s) leading to the Ca^++^-calmodulin interaction (see below for a discussion). We indeed find that if a Ca^++^ input (Fig. 3B, middle; see the Materials and Methods for details), fashioned after *Aplysia in vitro* experiments (Fig. 1A of [42]), arrives immediately before the transmitter, it accelerates the rise in Gα*/AC* concentration, as at this time point, Gα*/AC* formation is the dominant reaction. Consequently the area under the Gα*/AC* curve is increased. Assuming that the amount of cAMP production is proportional to the concentration of active adenylate cyclase, this translates into more cAMP production. If, however, Ca^++^ arrives once the transmitter has been reduced, it accelerates the fall of Gα*/AC* concentration (Fig. 3B, right), since at this time point, dissociation of Gα*/AC* is dominant. The area under the resulting Gα*/AC* curve is then smaller, meaning less cAMP production.

### Effect of the relative timing of the transmitter and Ca^++^

In the *Drosophila* learning experiment shown in Fig. 1, a control odour is given 210 s before electric shock; whereas a trained odour is paired with shock with varying inter-stimulus intervals (ISI). To simulate this experiment we represent the odour by the Ca^++^ input and the shock by the transmitter input. We neglect the very short time delays between the delivery of these stimuli and the resulting Ca^++^ influx into and transmitter release onto the Kenyon cells. Thus, in the control condition (Fig. 4, left), Ca^++^ arrives 210 s before the transmitter. We assume that the area under the resulting Gα*/AC* concentration curve reflects the total amount of cAMP produced. This can be thought of as the cAMP production in those Kenyon cells that are responsive to the control odour (i.e. ‘control’ Kenyon cells). During associative training (Fig. 4, right), Ca^++^ follows or leads the transmitter by a variable ISI. Again, the time integral of the respective Gα*/AC* concentration curve is taken as an estimate of cAMP production. Applied to the fly learning experiment in Fig. 1, this would be the amount of cAMP produced in those Kenyon cells that respond to the trained odour (i.e. ‘trained’ Kenyon cells). We plot the difference in cAMP production between the control condition and the associative training as percent of the control condition (Fig. 4, bottom: Percent associative effect). This reflects the test situation in the behavioural experiment in Fig. 1, where flies are given the choice between the control odour and the trained odour. Note that in the control condition, a non-zero amount of cAMP is produced; when applied to the learning experiment this would mean a basic amount of cAMP in all Kenyon cells, possibly causing a basic strengthening of their output. Indeed in flies, mere exposure to the shock modifies the olfactory behaviour; interestingly, the resulting non-associatively modified odour responses are less aversive upon loss of cAMP signalling or Kenyon cell function [47], [48].

Despite the overall simplicity of our approach, the simulation results (Fig. 4) agree strikingly well with the behavioural situation (Fig. 1). First, short negative or short positive ISIs result in negative associative effects; in other words, associative training with close Ca^++^ and transmitter pairing produces more cAMP than the control condition. Translating this to the learning experiment, *more* cAMP will be produced in the trained Kenyon cells than in the control Kenyon cells. Consequently, the output from the trained Kenyon cells to the downstream conditioned avoidance circuit will be strengthened more than that of the control Kenyon cells, resulting, at a choice situation, in relative avoidance of the trained odour (i.e. punishment learning). Next, for intermediate positive ISIs, the model produces positive associative effects, indicating *less* cAMP production during associative training than in the control condition. Applying this to the learning experiment, the output from the trained Kenyon cells to the downstream conditioned avoidance circuit will be strengthened less than the output from the control Kenyon cells. Consequently, given the choice between the two odours, the net behaviour will be conditioned approach towards the trained odour (i.e. relief learning). Finally, for very large (positive as well as negative) ISIs, the model shows no associative effect, as in the behavioural setting.

Other features of the simulation results are also reminiscent of the behavioural data in Fig. 1. First, the negative associative effect is larger than the positive associative effect. Second, the strongest negative associative effects are found when the onset of Ca^++^ precedes that of the transmitter; overlapping onsets result in a less pronounced negative associative effect. Note that, in behaviour, even an ISI of −45 s supports learning (Fig. 1); whereas in the model, negative ISIs longer than 5 s are not effective; this discrepancy is likely due to the properties of the Ca^++^ input in the present simulation (see Fig. 8 for a detailed analysis). Finally, in both behaviour and model, the strongest positive associative effects are obtained when the odour or Ca^++^ closely follows the offset of the shock or the transmitter.

Even with a single training trial, the negative and positive associative effects respectively reach up to ∼16% and 6% of the control, measured at the level of cAMP production. More intense Ca^++^ inputs (see Fig. 8 for details) and repetitive training will boost these effects significantly, as will the high amplification factors often seen in signal transduction cascades [49].

### Relationship between the adenylate cyclase dynamics and the associative effects

We next test how the agreement between model and behavioural data is influenced by changes in key model parameters. To this end, we first vary the rate constants for Gα*/AC* formation and dissociation (k_5_ and k_-5_). The dynamics of adenylate cyclase activation/deactivation (Fig. 5A) dictates both the ISI-dependency and the size of the associative effects (Figs. 5B1 and 5B2). Particularly, the duration of the rising and the falling phases of active adenylate cyclase concentration determine the window of ISI values appropriate for the negative and the positive associative effects, respectively. The sizes of the associative effects also depend on the dynamics of active adenylate cyclase concentration; intermediate speeds for build up and decay are best suited (see the legend of Fig. 5 for details). Notably, both the adenylate cyclase dynamics (Fig. 5A) and the associative effects (Figs. 5B1 and 5B2) remain stable over more than five orders of magnitude of the formation rate constant; whereas changes in the dissociation rate constant have much stronger influence.

**Figure 6:**
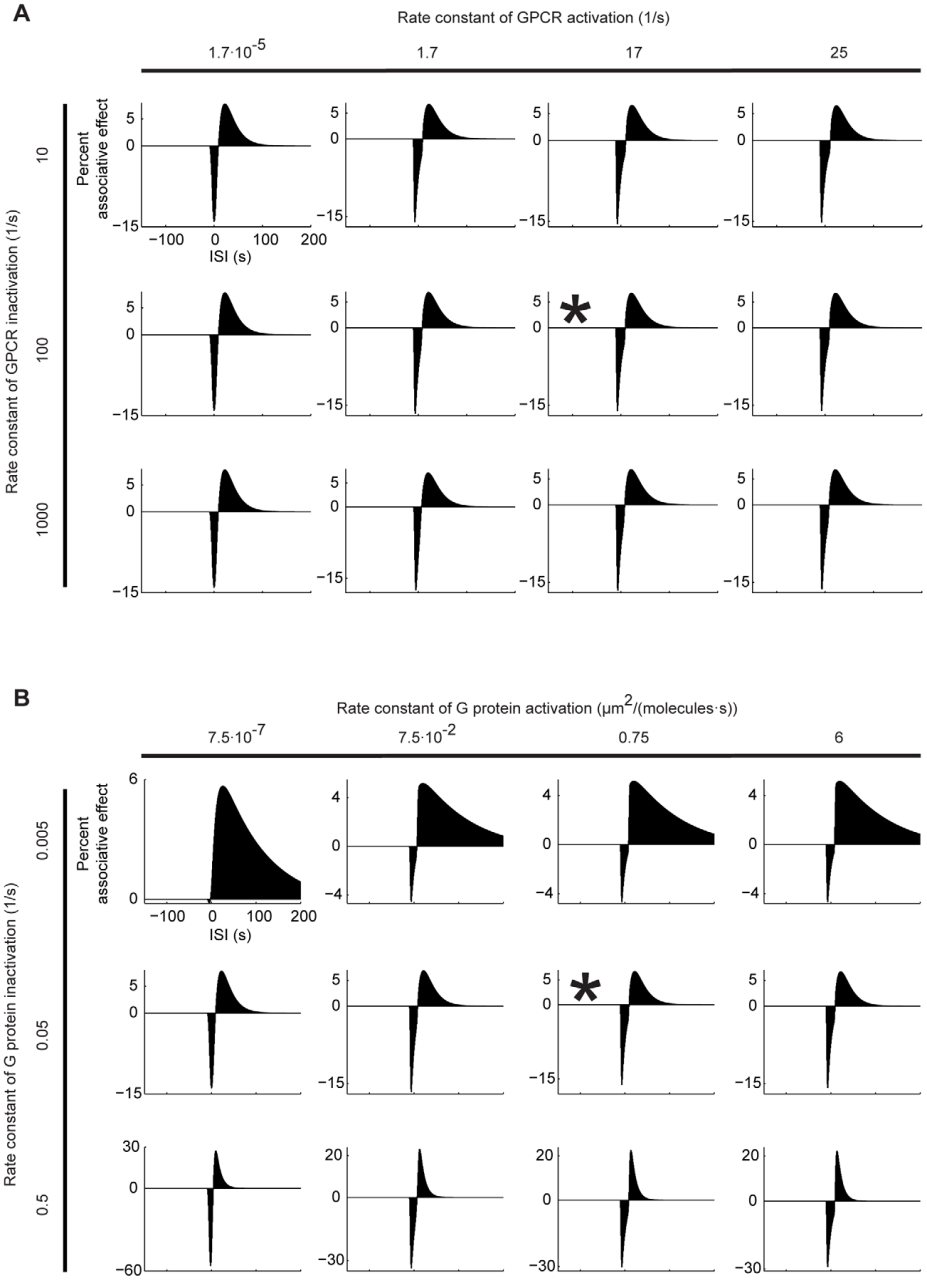
Dependence upon the activation and inactivation rate constants of GPCR and G protein. The percent associative effect is shown as a function of the ISI, as detailed in Fig. 4. Asterices mark the default conditions. A. Varying the rate constants for GPCR activation and inactivation hardly affects the size, or the ‘shape’ of the associative effects. B. Varying the rate constant of G protein activation also has nearly no bearings on the associative effects. As for the rate constant for G protein inactivation, higher values result in overall larger associative effects; this is because, both the rise and the fall of active adenylate cyclase concentration become moderately faster (not shown, see the legend of Fig. 5 for a more detailed explanation). In both (A) and (B), increasing the respective forward rate constants beyond the depicted range immediately recruits all available adenylate cyclase molecules, precluding any effect of Ca^++^ and thus any associative effect (not shown).

The associative effects are influenced little by varying the rate constants of GPCR (Fig. 6A) or G protein (Fig. 6B) activation and deactivation within a certain range. But when the respective forward rate constants are increased beyond the shown values, the associative effects abruptly decrease (see the legend of Fig. 6 for a detailed explanation). These findings agree with a previous, more systematical sensitivity-analysis [50] of the model proposed by Rospars et al. [46].

### Effects of the duration and intensity of the transmitter

To what extent do the observed associative effects depend on the specific properties of the inputs used?

**Figure 7:**
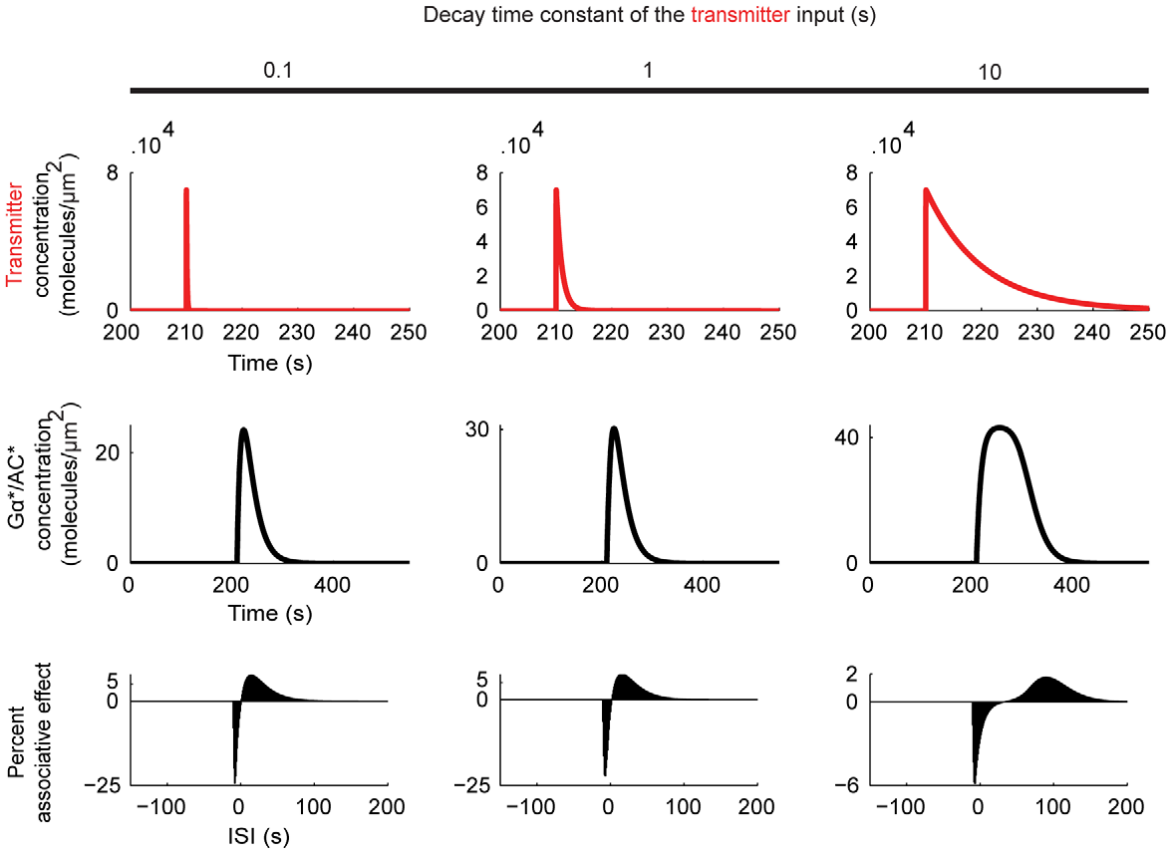
Influence of the transmitter duration. With a fixed Ca^++^ input, three different transmitter inputs are tested (top). They are all initiated at 210 s, rise to a peak of 7·10^4^ molecules/µm^2^ within 40 ms after the onset, but decay with different time constants as indicated above the panels. We plot the resulting adenylate cyclase dynamics (middle) and the ISI-dependent associative effects (bottom). In terms of the percent sizes of associative effects, changing the transmitter decay time constant from 0.1 to 1 (the first two cases) hardly makes a difference. A slower decaying transmitter input (the last case) broadens the dynamics of adenylate cyclase activation/deactivation, resulting in much higher cAMP production in the control condition; thus, the percent associative effects remain small. As for the ISI-dependence of the associative effects, short transmitter inputs (the first two cases) give good fits to the situation in Fig. 1; when a slower decaying transmitter input is used (the last case), the positive associative effect only occurs for large positive ISIs, due to the broadened adenylate cyclase activation/deactivation dynamics.

We first study the effect of changes in the duration of the transmitter (Fig. 7), keeping the Ca^++^ input the same as in the previous experiments. For a fixed rise time of the transmitter, increasing its decay time constant from 0.1 s to 1 s hardly changes the size of the associative effects (Fig. 7, the first two cases). A more slowly decaying transmitter input on the other hand, due to a much higher control level of cAMP production, allows only for smaller percent associative effects (Fig. 7, the last case). A corresponding effect of shock duration on the strength of learning remains to be probed for in fly learning experiments. As for the ISI-dependence of the associative effects, short transmitter inputs give good fit to the behavioural situation in Fig. 1 (Fig. 7, the first two cases). For more slowly decaying transmitter inputs, the positive associative effect only occurs for longer ISIs due to the broadened dynamics of adenylate cyclase activation/deactivation (Fig. 7, the last case). Quantitatively, we cannot provide a detailed comparison between these effects and those found at the behavioural level, since the dynamics of dopamine availability in the synaptic cleft upon shock stimulation is not known. It is however noteworthy that also in *Drosophila* behavioural experiments shock duration affects the window of ISIs appropriate for relief learning. For example, in Fig. 1, the shock lasts for 15 s; accordingly, relief learning is possible with ISIs longer than 15 s. For a 1.5s-long shock stimulus, however, an ISI of 2 s already supports relief learning (Fig. 8C of [17]). This invites a more systematic behavioural analysis of the effect of shock duration on relief learning.

**Figure 8:**
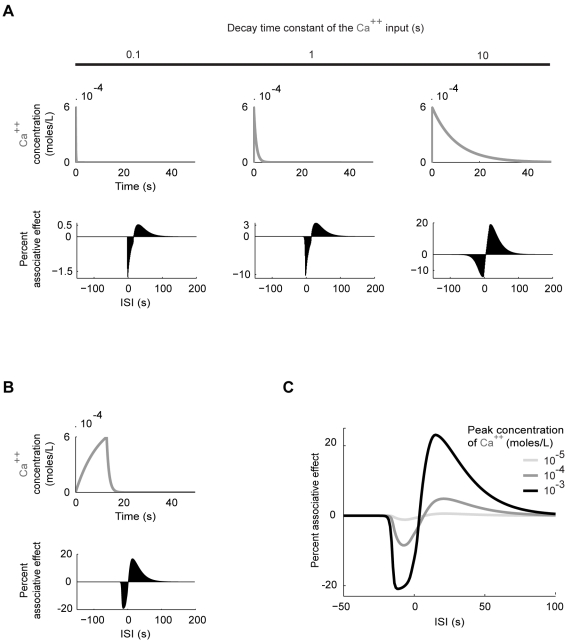
Influence of Ca^++^ duration and intensity. Complementing the analysis shown in Fig. 7 we now vary the Ca^++^ input while keeping the transmitter input fixed. In all three examples shown in (A), the Ca^++^ input rises to a peak of 6·10^−4^ moles/L within 40 ms after the Ca^++^ onset, but decays with different time constants, chosen as 0.1 s, 1 s and 10 s (A, top). In this scenario, the associative effects increase with increasing Ca^++^ duration (A, bottom). In addition, a large decay constant causes a long tail of the Ca^++^ input that enables negative associative effects for longer ISIs (A, the last case). In (B) we provide an exemplary Ca^++^ input (B, top) which gives good fit to the behavioural results in Fig. 1 in terms of the ISI-dependency of the associative effects but not in terms of their sizes relative to each other (B, bottom). In this case, the Ca^++^ concentration rises to a peak of 6·10^−4^ moles/L within 13 s after the onset, comparing well with the 15s- long odour presentation in Fig. 1. Note that the best negative associative effect occurs with ISI = −13 s, similar to the behavioural situation in Fig. 1. Finally, in (C), we study the effects of the intensity of the Ca^++^ input. We fix the transmitter input and use the Ca^++^ input depicted in (B), but scaled up and down by one order of magnitude. The intensity of Ca^++^ strongly influences the sizes of both the negative and the positive associative effects; the balance between the two is however somewhat compromised with increasing Ca^++^ intensity.

To test for the effects of varying the transmitter intensity, we use the intermediate time course shown in Fig. 7 and keep the Ca^++^ input as in the previous simulations. Scaling the transmitter input up and down over more than 10 orders of magnitude leaves the associative effects largely unchanged, both in terms of their percent size and their ISI-dependencies (data not shown). Only, unrealistically large transmitter inputs (≥10^7^ molecules/µm^2^), immediately activate all the available adenylate cyclase; this abolishes the possibility of modulation by Ca^++^ and precludes any associative effect (data not shown). These findings only partially reflect the situation in the fruit fly learning experiments, where intermediate shock intensities work best [13], [51].

### Effect of Ca^++^duration and intensity

In Fig. 1, given a 15s-long odour presentation, even an ISI of −45 s supports punishment learning. When adhesion of residual odour substance to the experimental setup is excluded, a 10s-long odour presentation enables punishment learning with an ISI of up to −25 s [52]. That is, a brief gap between the offset of the odour and the onset of the shock is readily tolerated. As an attempt to account for such ‘trace conditioning’ in our model, we vary the decay time constant of the Ca^++^ input, keeping constant its rise time and peak (Fig. 8A, top). In fact, implementing the biochemical steps of Ca^++^-calmodulin interaction would likely have the same effect (Fig. 1 of [53]). In any case, more slowly decaying Ca^++^ inputs lead to larger associative effects (Fig. 8A, bottom); and enable longer ISIs to lead to negative associative effects (Fig. 8A, the last case). As exemplified in Fig. 8B, the shape of the Ca^++^ input is indeed a critical parameter for reproducing the behavioural situation in Fig. 1 (see the respective figure legend for details). In short, a long tail of odour-induced Ca^++^ (or Ca^++^-calmodulin complex) increase in the Kenyon cells could bridge over at least part of the temporal gap between odour offset and shock onset. This could then be used by the Ca^++^-calmodulin-dependent adenylate cyclase or likely also other signalling molecules [54] to enable ‘trace conditioning’. Studies using genetically encoded Ca^++^ sensors to monitor the Kenyon cell odour responses neither rule out nor confirm the existence of such long tails in the Ca^++^ concentration [22]–[24].

Next, we look at the effects of the intensity of the Ca^++^ input. To investigate a scenario that mimics the behavioral situation as closely as possible, we use Ca^++^ inputs shaped as in Fig. 8B. We scale their size up or down by one order of magnitude. As shown in Fig. 8C, this strongly influences both the negative and the positive associative effects. In the fruit fly, too, learning is typically improved with increasing odour concentration; beyond a certain concentration, however, further increase deteriorates learning [13], which is not explained by our model.

### Alternative model for the adenylate cyclase regulation

**Figure 9:**
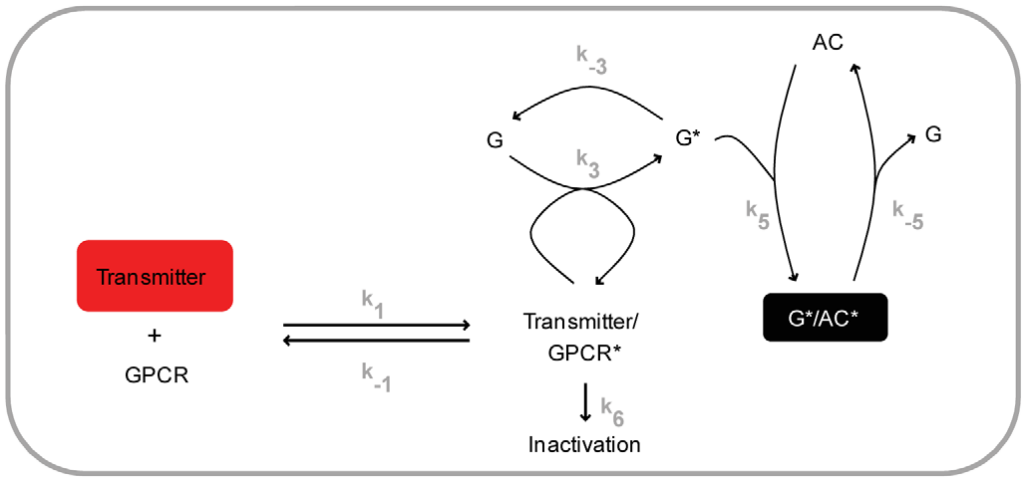
An alternative model for adenlyate cyclase regulation by the transmitter. To complement our main analysis based on the model adapted from [46] and shown in Fig. 3A, we finally use a simpler model variant [45]. Here, the transmitter reversibly binds to its respective G protein coupled receptor (GPCR) to form an active complex (Transmitter/GPCR*). This complex then dissociates, or it interacts with the G protein (G) to activate it (G*). The trimeric nature of the G protein is ignored (compare with Fig. 3A). G* on the one hand spontaneously deactivates (G), on the other hand it reversibly interacts with the adenylate cyclase (AC) to form an enzymatically active complex (G*/AC*), which serves as the system's output. The effect of Ca^++^ is implemented the same way as in Fig. 3A.

Finally, we test the generality of our results using an alternative model for the regulation of the adenylate cyclase by the transmitter [45]. This model (Fig. 9) includes only a single biochemical step for the GPCR activation and it ignores the trimeric nature of the G protein. In addition to its reduced complexity (i.e. five instead of nine differential equations), it differs from the first model (Fig. 3A) in terms of the initial concentrations of the molecules, as well as the reaction rate constants (see Materials and Methods for details).

**Figure 10:**
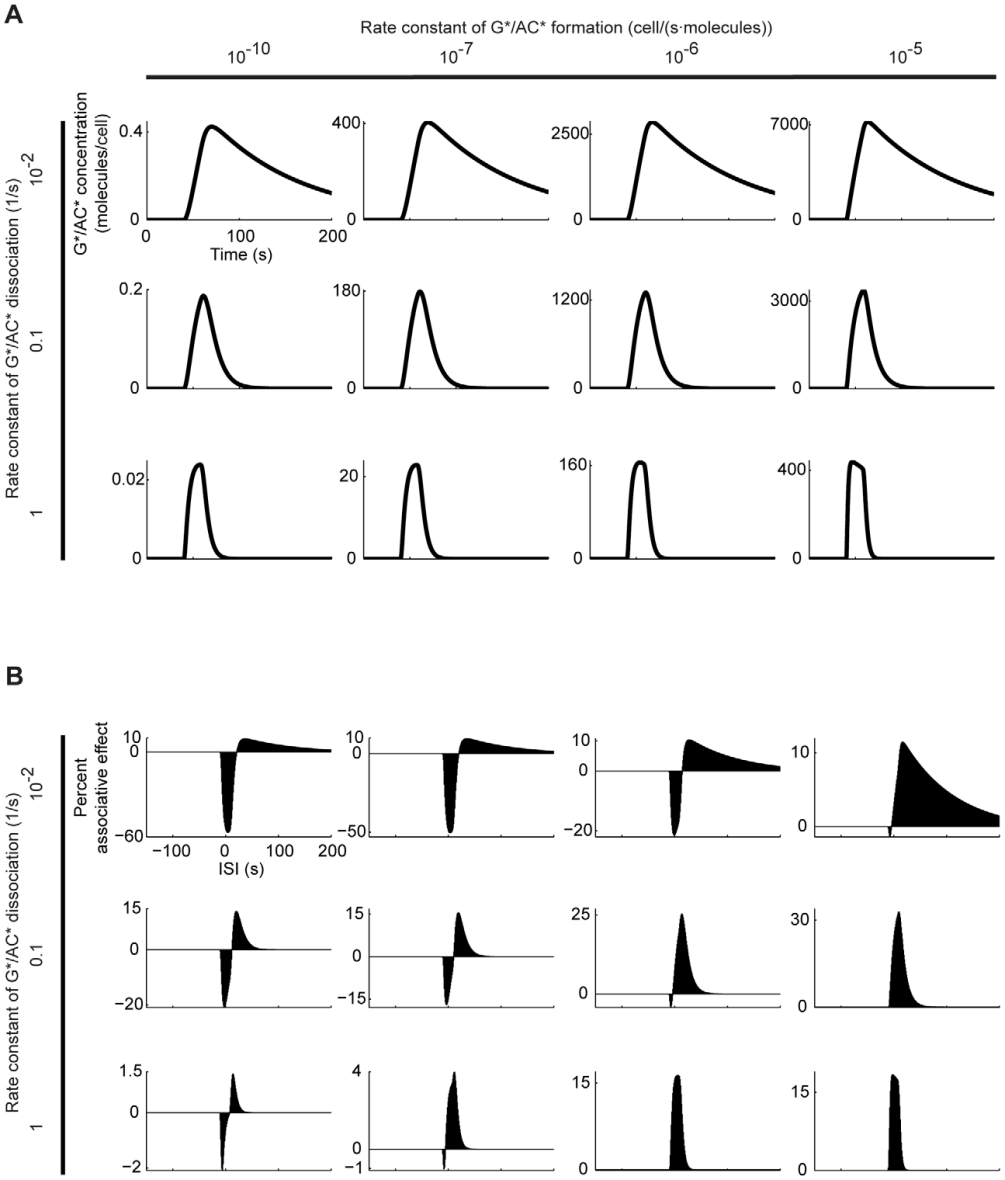
Alternative model: Influence of G*/AC* formation and dissociation rate constants. A. We stimulate the alternative model based on [45] with a transmitter input (details as in Fig. 3B) and plot the time course of the resulting G*/AC* concentration. B. Repeating the experiment in Fig. 4, we plot the percent associative effect as a function of the ISI. Comparison with Fig. 5 shows that despite their various differences both models generate rather similar associative effects.

In response to a transmitter input, the alternative model generates time courses for the active adenylate cyclase concentration (Fig. 10A) and associative effects (Fig. 10B) whose salient features are strikingly similar to those of the first model (Fig. 5). Most importantly, the simplified model also clearly shows opposing associative effects that depend in the same qualitative manner on event timing and the adenylate cyclase dynamics.

Note that the two models we use are adapted from two different systems (i.e., olfactory transduction in moth [46] and actin polymerization in human neutrophils [45], respectively) and thus the parameter estimates come from different methods, processes and species. Having reconciled these, we are confident that our results capture the generic properties of Ca^++^-calmodulin-sensitive adenylate cyclase regulation. We believe that this cross-species approach we use strengthens the proof of concept that the reaction dynamics of adenylate cyclase signalling could explain the effect of event timing on associative learning.

## Discussion

Event timing critically affects associative learning. Fruit flies, for example, learn an odour as a signal for punishment or relief, depending on whether it precedes or follows shock during training (Fig. 1) [11], [13]–[17]. We suggest a simple biochemical explanation for these two opposing kinds of learning. During punishment training, a Ca^++^-calmodulin-sensitive adenylate cyclase in the Kenyon cells seems to detect the convergence of the odour and the shock signals (Fig. 2) [30]–[35] (see also [55] for a similar mechanism in striatal medium spiny neurons). Based on biochemical data from *Aplysia* [41]–[43], we implement a model where shock-induced transmitter activates the adenylate cyclase and the underlying reaction dynamics are bi-directionally regulated by odour-induced Ca^++^, depending on the relative timing of the two inputs (Fig. 3). Using this model, we simulate the key fruit fly learning experiment for the effect of event timing (Fig. 1). To mimic the situation in the control Kenyon cells, we use Ca^++^ and transmitter inputs that are sufficiently separated in time, and assume that the resulting cAMP production will strengthen the output of these cells to the conditioned avoidance circuits to a certain level. To simulate the situation in the trained Kenyon cells, we use various inter-stimulus intervals (ISI) between the Ca^++^ and the transmitter. In this setting, the equivalent of punishment training leads to more cAMP than the control level (Fig. 4) so that the output of the trained Kenyon cells will be strengthened more than that of the control Kenyon cells, resulting in avoidance of the trained odour in a choice situation. The equivalent of relief training, in turn, results in a cAMP production below the control level (Fig. 4); consequently, the output of the trained Kenyon cells will remain weaker than that of the control Kenyon cells, resulting in net approach to the trained odour. Despite its simple biochemical formulation, the model also recapitulates other salient features of punishment and relief learning (Fig. 4). This agreement between the simulation- and the behavioural data is robust with respect to changes in various model parameters within reasonably wide ranges (Figs. 5 and 6). Given the effects observed beyond these ranges however, it may be interesting to experimentally manipulate reaction rate constants, e.g., by changing ambient temperature, to then see the effects on the behavioural ISI-learning function. Importantly, our conclusions also hold for a rather different model for transmitter-mediated adenylate cyclase activation (Figs. 9 and 10).

The associative effects we report in Fig. 4 reach up to ∼16% of the control condition; a stronger Ca^++^ input boosts these effects significantly (Fig. 8C); also, repetition of training will result in a cumulative increase. In addition, these effects will most likely be amplified through the downstream signal transduction cascade [49]. Note that a previous model based on spike-timing-dependent plasticity reports up to only ∼0.7% change in synaptic strength in a single training trial (Fig. 1C of [18]), despite assuming unrealistically strong odour-induced activity. Experimentally, e.g., in vertebrate brain slices, no less than 20 pre-post synaptic action potential pairings are necessary to obtain only ∼10% potentiation of synaptic strength [56]. Given these, the sizes of the associative effects we report seem reasonable. Critically, the quantitative relationship between synaptic plasticity and behavioural plasticity has not been characterized with respect to *Drosophila* olfactory learning; whereas few studies exist in other systems, e.g., [57] reports 10–20% strengthening in hippocampal synapses upon behavioural training. In general, the question of how much change in synaptic strength is *required* for making a difference in behaviour, is open.

The present model uses the amount of cAMP production as output. Clearly, much happens in reality between this step and the synaptic plasticity underlying learning. Implementing the following stages of signal transduction (e.g., activation of the cAMP-dependent protein kinase (PKA), phosphorylation of Synapsin [58]) may help understanding key features of associative learning, other than its sensitivity to event timing. For example, in the honey bee antennal lobe, a single olfactory reward training trial transiently activates PKA; repetitive training on the other hand results in prolonged PKA activation, which may be important for the formation of long-term memory [59] (see [60] for a computational model relying on this mechanism). Also, degradation of cAMP [35] and de-phosphorylation of key downstream proteins [61] is likely critical for restricting the effects of learning both during training and thereafter. All these downstream processes can be added to the model to explain the dynamics of memory acquisition or decay. In the current study however we focus on the effect of event timing on learning and provide a proof of concept that bi-directional regulation of adenylate cyclase can be the underlying mechanism. As a next step, one should experimentally test for a role of the Ca^++^-calmodulin-sensitive adenylate cyclase, *rutabaga* in relief learning, using the available genetic tools, e.g., loss of function mutations [30], [32], [33], RNAi-knockdown [62]. Also the role of dopaminergic signalling in relief learning remains open. Blocking the neuronal output from two different, incomplete sets of dopaminergic neurons leaves relief learning intact [16]; however, given the caveats of the genetic techniques used in the respective study, the complementary approach of interfering with dopamine receptor function using genetic [27], [63] and pharmacological [64] tools seems warranted. Note that for punishment learning, both the adenylate cyclase- and the dopamine roles are better established [25]–[33].

A previous model by Drew and Abbott [18] suggests that punishment learning strengthens the Kenyon cell output, whereas relief learning has a weakening effect, so that opposite kinds of conditioned behaviour result. As key mechanism the authors implement spike-timing-dependent plasticity (STDP) at the Kenyon cell output synapses [65], [66]. To bridge the gap in time scales between STDP and behavioural event timing effects, they need to assume high and slowly decaying spiking activity in the Kenyon cells and postsynaptic neurons, following odour and shock, respectively. As these assumptions are experimentally not fulfilled [19]–[21], this particular, STDP-based model does not seem appropriate for olfactory learning in the fruit fly. This does however not exclude a role for STDP in insect olfactory learning: In the locust, specific Kenyon cell output synapses seem to be ‘tagged’ by the occurrence of temporally adjunct pre- and post-synaptic action potentials, mimicking the situation during odour presentation; only these tagged synapses are then modified upon delivery of a delayed neuromodulator [66]. Such a process could underlie punishment learning, including ‘trace conditioning’; it can however not readily account for relief learning.

Both in Drew and Abbott's STDP-based model [18] and in the present adenylate cyclase-based model, punishment and relief training act on the same Kenyon cell output to the same downstream circuit, but in opposite ways. This scenario readily accounts for the observed diametrically opposite conditioned behaviours, i.e. avoidance *vs.* approach [11], [13]–[17]. Further investigation into the repertoire of conditioned behaviours after punishment and relief training may well render this scenario short, e.g., if punishment learning can modulate kinds of behaviour that relief learning leaves unaffected and *vice versa*. In that case, an alternative scenario could be that punishment and relief learning strengthen the output from two distinct sets of Kenyon cells which redundantly encode the trained odour, but receive different kinds of reinforcement signal and send their output to different downstream circuits. In a related scenario, punishment and relief memory traces would be laid down within the same Kenyon cells, but at distinct sub-cellular sites, which receive different reinforcement signals and send output to different downstream circuits. In either case, it is not known how the reinforcement signal for relief is implemented at the neuronal level [16]. Finally, with respect to all scenarios discussed, the role of the Kenyon cells in relief learning awaits testing. Note that for punishment learning, this role is well-established [25], [31]–[33].

To summarize, further experiments on the molecular, cellular and behavioural level are needed to elucidate the mechanism of relief learning. The present computational study may guide this process in that it identifies one plausible candidate scenario. More generally, our approach shows that even a simple biochemical process may help explain a non-trivial behavioural observation, such as the bi-directional effect of event timing on associative learning.

## Materials and Methods

All simulations were done in MATLAB 7 (Mathworks, Natick, USA) on a PC. Except in Figs. 5B2, 8B and 8C, the differential equations were solved using the forward Euler method, where the time-dependent inputs and dynamical variables were discretized at 0.001 s. Variations of the temporal step size showed that this approach yielded a faithful yet simple numerical representation of the dynamics. In Figs. 5B2, 8B and 8C, we used the ordinary differential equation solver ode15 s, provided by MATLAB.

### Regulation of the adenylate cyclase by the transmitter and Ca^++^

We implemented two alternative models for the regulation of the adenylate cyclase by the transmitter and Ca^++^. The first model was adapted from a previous model of G-protein-mediated insect olfactory signal transduction [46]. The alternative model was adapted from an implementation of G-protein signalling in actin polymerization in human neutrophils [45]. In what follows, we present in detail the first model; the alternative model is briefly explained at the end.

The transmitter (Tr) was the primary input to the model, as sketched in Fig. 3A. Unless stated otherwise, the time course of the Tr concentration was fashioned after biochemical experiments performed in *Aplysia*. To this end, we extracted the time-dependent serotonin concentration from Fig. 1A of [42] and used linear interpolation to generate the additional data points required for the simulations. Numerical values were converted from µmoles/L to molecules/µm^2^ using a conversion factor.
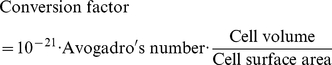
(1)Avogadro's number is 6.02·10^23^ molecules/mol and, following [46], the cell volume was 2600 µm^3^ and cell surface area was 426 µm^2^, leading to a conversion factor of ∼3700 molecules·µm/mol. The resulting Tr concentration reached a peak value of 6.7·10^4^ molecules/µm^2^ within ∼7 s and decayed back to zero within ∼18 s after stimulus onset.

For the simulations depicted in Fig. 7, the Tr concentration over time was taken as
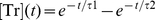
(2)To cover different decay courses, the time constant τ1 was chosen as 0.1 s, 1 s and 10 s, respectively; with τ2 = 0.01 s, the peak concentration was reached within ∼40 ms after transmitter onset. The resulting concentrations were normalized such that the peak was 7·10^4^ molecules/µm^2^ in each case. For varying Tr intensity, we used the time constants τ1 = 1 s and τ2 = 0.01 s and up- and down-scaled the respective function by division.

In each experiment, the desired Tr concentration time course was initiated at the specified point in time. In Figs. 3B and 4, for plotting reasons, concentrations were normalized relative to their peak values. For molecules other than Ca^++^ and Tr, concentrations were initiated with the values specified in Table 1.

**Table 1:** Components and initial concentrations for the first model.

Abbreviation	Molecule	Initial concentration (molecules/µm^2^)
GPCR	G protein coupled receptor	6000
Tr/GPCR	Complex of Tr and GPCR	0
GPCR*	Activated GPCR	0
Gαβγ	Trimeric G protein	1000
Gβγ	G protein β- and γ- subunits	0
Gα*	Active Gα	0
Gα	Inactive G protein α-subunit	0
AC	Adenylate cyclase	500
Gα*/AC*	Complex of Gα* and activated AC	0

All values were chosen according to [46] and were estimates from moth olfactory transduction (see [46] for further references).

The concentration of each kind of molecule was then updated according to the respective equation, below.
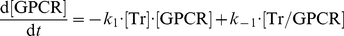
(3)

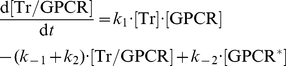
(4)

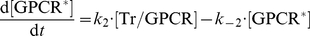
(5)

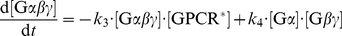
(6)

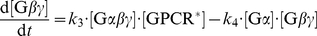
(7)

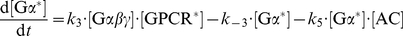
(8)

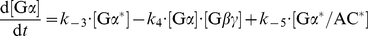
(9)

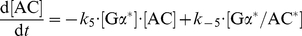
(10)

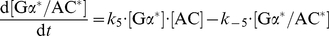
(11)In these equations, the reaction rate constants (k) took the values listed in Table 2.

**Table 2:** Rate constants of the reactions for the first model.

Rate constant	Reaction	Value	Unit
k_1_	Formation of the Tr/GPCR complex	5.6·10^−5^	µm^2^/(molecules·s)
k_-1_	Dissociation of the Tr/GPCR complex	8	1/s
k_2_	Activation of GPCR	17	1/s
k_-2_	Inactivation of GPCR	100	1/s
k_3_	Dissociation of Gαβγ into Gα* and Gβγ	0.75	µm^2^/(molecules·s)
k_-3_	Deactivation of Gα* to Gα	0.05	1/s
k_4_	Reassembly of Gα and Gβγ into Gαβγ	2	µm^2^/(molecules·s)
k_5_^base-line^	Formation of the Gα*/AC* complex	10^−5^	µm^2^/(molecules·s)
k_-5_^base-line^	Dissociation of the Gα*/AC* complex	0.1	1/s

Apart from k_5_ and k_-5_, all values were chosen according to [46]. Thus, k_1_, k_-1_, k_2_, k_-2_ were estimates from moth olfactory transduction or vertebrate phototransduction (see [46] for further references). For the parameters k_5_ and k_-5_ (see also Eqs. 13 and 14), the listed base-line values were chosen to mimic the experimentally measured dynamics of adenylate cyclase activation/deactivation in response to transmitter [42], for a detailed sensitivity-analysis, see Fig. 5A. k_5_ and k_-5_ were sensitive to Ca^++^ (Eqs. 13 and 14).

Ca^++^ was the second input to the model. Unless stated otherwise, its time-dependent concentration was modeled according to data from biochemical experiments carried out in *Aplysia* (Fig. 1A of [42]), using linear interpolation. The resulting Ca^++^ concentration started to rise at ∼4.5 s, reached a peak value of 5.6·10^−4^ moles/L within 6 s and decayed back to zero within ∼8.5 s after stimulus onset. For Fig. 8A, the Ca^++^ concentration was calculated according to the Eq. (2), and then normalized such that the peak value was 6·10^−4^ moles/L. In Figs. 8B and 8C, the Ca^++^ concentration over time was taken as
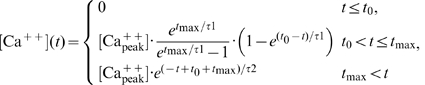
(12)[Ca^++^_peak_], the maximum value of [Ca^++^], was taken as 6·10^−4^ moles/L in Fig. 8B and was varied as shown in Fig. 8C. t_0_ was the onset of the Ca^++^ input; t_max_ = 13 s was the time it took the [Ca^++^] to reach its maximum; τ1 = 10 s and τ2 = 1 s were the time constants of [Ca^++^] rise and fall, respectively.

In order to account for the findings in *Aplysia* [41]–[43] (see Results for details), we assumed Ca^++^ to affect the reaction rate constants k_5_ and k_-5_ with a delay of 2.5 s so that k_5_ and k_-5_ became
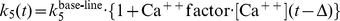
(13)

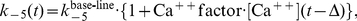
(14)where the time-dependent input [Ca^++^](t) is replaced by [Ca^++^](t-Δ) and Δ = 2.5 s. We used Ca^++^ factor = 10 000 L/(moles·s).

### Effect of event timing on the adenylate cyclase

The model system was stimulated with a transmitter input, as described above, delivered at time t = 210 s. For the control condition, a Ca^++^ input was given at t = 0 s. For the associative training, the Ca^++^ input was separated from the transmitter input with an inter-stimulus interval (ISI), which was varied across experiments between −150 s and 200 s in steps of 1 s, except in Fig. 5B2, where the range was −100 s to 200 s. Here, negative ISIs indicated that Ca^++^ preceded the transmitter; positive ISIs meant that Ca^++^ followed the transmitter. The timing of stimuli was fashioned after the behavioural experiment in Fig. 1. During the 550s- long simulation, the area under the Gα*/AC* concentration curve was taken as a measure of cAMP production. For each ISI, we then calculated the percent associative effect as
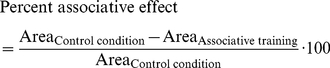
(15)Negative values thus indicated that associative training with the particular ISI resulted in more cAMP production than the control condition; positive values meant less cAMP production compared to control.

### Alternative model

The alternative model for the dual control of the adenylate cyclase by the transmitter (Tr) and Ca^++^ was based on [45] and is sketched in Fig. 9. The Tr and Ca^++^ concentrations were chosen according to the experiments performed in *Aplysia* (Fig. 1A of [42]), as already explained in the context of the first model, except that Tr concentration was measured in moles/L. Model components and initial concentrations are given in Table 3. The dynamical variables were updated according to the Eqs. (16) to (21) and the reaction rate constants are given in Table 4. The effect of Ca^++^ and the percent associative effect were defined as in the first model.
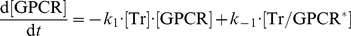
(16)

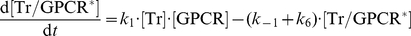
(17)

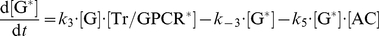
(18)

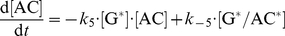
(19)

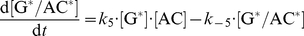
(20)

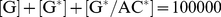
(21)


**Table 3:** Components and initial concentrations for the alternative model.

Abbreviation	Molecule	Initial concentration (molecules/cell)
GPCR	G protein coupled receptor	55 000
Tr/GPCR*	Complex of Tr and activated GPCR	0
G*	Activated G protein	0
AC	Adenylate cyclase	100 000
G*/AC*	Complex of G* and activated AC	0
G	G protein	100 000

Apart from the initial concentrations of AC and G*/AC*, values were as in [45] and thus estimates from neutrophil actin polymerization (see [45] for further references).

**Table 4:** Rate constants of the reactions for the alternative model.

Rate constant	Reaction	Value	Unit
k_1_	Formation of the Tr/GPCR* complex	8.4·10^7^	L/(moles·s)
k_-1_	Dissociation of the Tr/GPCR* complex	0.37	1/s
k_6_	Internalization of the Tr/GPCR* complex	0.0065	1/s
k_3_	Activation of G	10^−7^	cell/(molecules·s)
k_-3_	Deactivation of G	0.2	1/s

All parameter values were as in [45] and thus measured or estimates from neutrophil actin polymerization (see [45] for further references). k_5_ and k_-5_, which are not included in the table, changed upon stimulation with Ca^++^ (Eqs. 13 and 14); their base-line values were varied in Fig. 10.

## References

[1] Rescorla RA (1988). Behavioral studies of Pavlovian conditioning.. Annu Rev Neurosci.

[2] Solomon RL, Corbit JD (1974). An opponent-process theory of motivation. I. Temporal dynamics of affect.. Psychol Rev.

[3] Wagner AR, Spear NE, Miller RR (1981). SOP: A model of automatic memory processing in animal behavior.. Information Processing in Animals: Memory Mechanisms.

[4] Sutton RS, Barto AG, Gabriel M, Moore J (1990). Time-derivative models of pavlovian reinforcement.. Learning and computational neuroscience: Foundations of adaptive networks.

[5] Chang RC, Blaisdell AP, Miller RR (2003). Backward conditioning: mediation by the context.. J Exp Psychol Anim Behav Process.

[6] Moscovitch A, LoLordo VM (1968). Role of safety in the Pavlovian backward fear conditioning procedure.. J Comp Physiol Psychol.

[7] Plotkin HC, Oakley DA (1975). Backward conditioning in the rabbit (Oryctolagus cuniculus).. J Comp Physiol Psychol.

[8] Maier SF, Rapaport P, Wheatley KL (1976). Conditioned inhibition and the UCS-CS interval.. Anim Learn Behav.

[9] Hellstern F, Malaka R, Hammer M (1998). Backward inhibitory learning in honeybees: a behavioral analysis of reinforcement processing.. Learn Mem.

[10] Britton G, Farley J (1999). Behavioral and neural bases of noncoincidence learning in Hermissenda.. J Neurosci.

[11] Tanimoto H, Heisenberg M, Gerber B (2004). Experimental psychology: event timing turns punishment to reward.. Nature.

[12] Andreatta M, Muhlberger A, Yarali A, Gerber B, Pauli P (2010). A rift between implicit and explicit conditioned valence in human pain relief learning.. Proc Biol Sci.

[13] Yarali A, Niewalda T, Chen Y-c, Tanimoto H, Duerrnagel S (2008). ‘Pain relief’ learning in fruit flies.. Animal Behavior.

[14] Khurana S, Abu Baker MB, Siddiqi O (2009). Odor avoidance learning in the larva of Drosophila melanogaster.. J Biosci.

[15] Yarali A, Krischke M, Michels B, Saumweber T, Mueller MJ (2009). Genetic distortion of the balance between punishment and relief learning in Drosophila.. J Neurogenet.

[16] Yarali A, Gerber B (2010). A Neurogenetic Dissociation between Punishment-, Reward-, and Relief-Learning in Drosophila.. Front Behav Neurosci.

[17] Murakami S, Dan C, Zagaeski B, Maeyama Y, Kunes S (2010). Optimizing Drosophila olfactory learning with a semi-automated training device.. J Neurosci Methods.

[18] Drew PJ, Abbott LF (2006). Extending the effects of spike-timing-dependent plasticity to behavioral timescales.. Proc Natl Acad Sci U S A.

[19] Ito I, Ong RC, Raman B, Stopfer M (2008). Olfactory learning and spike timing dependent plasticity.. Commun Integr Biol.

[20] Murthy M, Fiete I, Laurent G (2008). Testing odor response stereotypy in the Drosophila mushroom body.. Neuron.

[21] Turner GC, Bazhenov M, Laurent G (2008). Olfactory representations by Drosophila mushroom body neurons.. J Neurophysiol.

[22] Wang Y, Guo HF, Pologruto TA, Hannan F, Hakker I (2004). Stereotyped odor-evoked activity in the mushroom body of Drosophila revealed by green fluorescent protein-based Ca2+ imaging.. J Neurosci.

[23] Yu D, Akalal DB, Davis RL (2006). Drosophila alpha/beta mushroom body neurons form a branch-specific, long-term cellular memory trace after spaced olfactory conditioning.. Neuron.

[24] Wang Y, Mamiya A, Chiang AS, Zhong Y (2008). Imaging of an early memory trace in the Drosophila mushroom body.. J Neurosci.

[25] Schwaerzel M, Monastirioti M, Scholz H, Friggi-Grelin F, Birman S (2003). Dopamine and octopamine differentiate between aversive and appetitive olfactory memories in Drosophila.. J Neurosci.

[26] Riemensperger T, Voller T, Stock P, Buchner E, Fiala A (2005). Punishment prediction by dopaminergic neurons in Drosophila.. Curr Biol.

[27] Kim YC, Lee HG, Han KA (2007). D1 dopamine receptor dDA1 is required in the mushroom body neurons for aversive and appetitive learning in Drosophila.. J Neurosci.

[28] Claridge-Chang A, Roorda RD, Vrontou E, Sjulson L, Li H (2009). Writing memories with light-addressable reinforcement circuitry.. Cell.

[29] Aso Y, Siwanowicz I, Bräcker L, Ito K, Kitamoto T (2010). Specific dopaminergic neurons for the formation of labile aversive memory.. Curr Biol.

[30] Livingstone MS, Sziber PP, Quinn WG (1984). Loss of calcium/calmodulin responsiveness in adenylate cyclase of rutabaga, a Drosophila learning mutant.. Cell.

[31] Connolly JB, Roberts IJ, Armstrong JD, Kaiser K, Forte M (1996). Associative learning disrupted by impaired Gs signaling in Drosophila mushroom bodies.. Science.

[32] Zars T, Fischer M, Schulz R, Heisenberg M (2000). Localization of a short-term memory in Drosophila.. Science.

[33] Blum AL, Li W, Cressy M, Dubnau J (2009). Short- and long-term memory in Drosophila require cAMP signaling in distinct neuron types.. Curr Biol.

[34] Tomchik SM, Davis RL (2009). Dynamics of learning-related cAMP signaling and stimulus integration in the Drosophila olfactory pathway.. Neuron.

[35] Gervasi N, Tchenio P, Preat T (2010). PKA dynamics in a Drosophila learning center: coincidence detection by rutabaga adenylyl cyclase and spatial regulation by dunce phosphodiesterase.. Neuron.

[36] Séjourné J, Plaçais PY, Aso Y, Siwanowicz I, Trannoy S (2011). Mushroom body efferent neurons responsible for aversive olfactory memory retrieval in Drosophila.. Nat Neurosci.

[37] Lin AH, Cohen JE, Wan Q, Niu K, Shrestha P (2010). Serotonin stimulation of cAMP-dependent plasticity in Aplysia sensory neurons is mediated by calmodulin-sensitive adenylyl cyclase.. Proc Natl Acad Sci U S A.

[38] Abrams TW (1985). Activity-dependent presynaptic facilitation: an associative mechanism in Aplysia.. Cell Mol Neurobiol.

[39] Ocorr KA, Walters ET, Byrne JH (1985). Associative conditioning analog selectively increases cAMP levels of tail sensory neurons in Aplysia.. Proc Natl Acad Sci U S A.

[40] Abrams TW, Karl KA, Kandel ER (1991). Biochemical studies of stimulus convergence during classical conditioning in Aplysia: dual regulation of adenylate cyclase by Ca2+/calmodulin and transmitter.. J Neurosci.

[41] Yovell Y, Abrams TW (1992). Temporal asymmetry in activation of Aplysia adenylyl cyclase by calcium and transmitter may explain temporal requirements of conditioning.. Proc Natl Acad Sci U S A.

[42] Abrams TW, Yovell Y, Onyike CU, Cohen JE, Jarrard HE (1998). Analysis of sequence-dependent interactions between transient calcium and transmitter stimuli in activating adenylyl cyclase in Aplysia: possible contribution to CS–US sequence requirement during conditioning.. Learn Mem.

[43] Onyike CU, Lin AH, Abrams TW (1998). Persistence of the interaction of calmodulin with adenylyl cyclase: implications for integration of transient calcium stimuli.. J Neurochem.

[44] Lin AH, Onyike CU, Abrams TW (1998). Sequence-dependent interactions between transient calcium and transmitter stimuli in activation of mammalian brain adenylyl cyclase.. Brain Res.

[45] Adams JA, Omann GM, Linderman JJ (1998). A mathematical model for ligand/receptor/G-protein dynamics and actin polymerization in human neutrophils.. J Theor Biol.

[46] Rospars JP, Lucas P, Coppey M (2007). Modeling the early steps of transduction in insect olfactory receptor neurons.. Biosystems.

[47] Preat T (1998). Decreased odor avoidance after electric shock in Drosophila mutants biases learning and memory tests.. J Neurosci.

[48] Acevedo SF, Froudarakis EI, Tsiorva A-A, Skoulakis EMC (2007). Distinct neuronal circuits mediate experience-dependent, non-associative osmotactic responses in Drosophila.. Mol Cell Neurosci.

[49] Alberts B, Johnson A, Levis J, Raff M, Roberts K (2002). Molecular Biology of the Cell. 4th ed.

[50] Halnes G, Ulfhielm E, Ljunggren EE, Kotaleski JH, Rospars J-P (2009). Modelling and sensitivity analysis of the reactions involving receptor, G-protein and effector in vertebrate olfactory receptor neurons.. J Comput Neurosci.

[51] Tully T, Quinn WG (1985). Classical conditioning and retention in normal and mutant Drosophila melanogaster.. J Comp Physiol A.

[52] Galili DS, Lüdke A, Galizia CG, Szyszka P, Tanimoto H (2011). Olfactory trace conditioning in Drosophila.. J Neurosci.

[53] Dougherty DP, Wright GA, Yew AC (2005). Computational model of the cAMP-mediated sensory response and calcium-dependent adaptation in vertebrate olfactory receptor neurons.. Proc Natl Acad Sci U S A.

[54] Shuai Y, Hu Y, Qin H, Campbell RA, Zhong Y (2011). Distinct molecular underpinnings of Drosophila olfactory trace conditioning.. Proc Natl Acad Sci U S A.

[55] Lindskog M, Kim M, Wikström MA, Blackwell KT, Kotaleski JH (2006). Transient calcium and dopamine increase PKA activity and DARPP-32 phosphorylation.. PLoS Comp Biol.

[56] Zhang J, Lau P, Bi G (2009). Gain in sensitivity and loss in temporal contrast of STDP by dopaminergic modulation at hippocampal synapses.. Proc Natl Acad Sci U S A.

[57] Whitlock JR, Heynen AJ, Shuler MG, Bear MF (2006). Learning induces long-term potentiation in the hippocampus.. Science.

[58] Michels B, Chen YC, Saumweber T, Mishra D, Tanimoto H (2011). Cellular site and molecular mode of synapsin action in associative learning.. Learn Mem.

[59] Muller U (2000). Prolonged activation of cAMP-dependent protein kinase during conditioning induces long-term memory in honeybees.. Neuron.

[60] Aszodi A, Muller U, Friedrich P, Spatz HC (1991). Signal convergence on protein kinase A as a molecular correlate of learning.. Proc Natl Acad Sci U S A.

[61] Pagani MR, Oishi K, Gelb BD, Zhong Y (2009). The phosphatase SHP2 regulates the spacing effect for long-term memory induction.. Cell.

[62] Pan Y, Zhou Y, Guo C, Gong H, Gong Z (2009). Differential roles of the fan-shaped body and the ellipsoid body in Drosophila visual pattern memory.. Learn Mem.

[63] Selcho M, Pauls D, Han KA, Stocker RF, Thum AS (2009). The role of dopamine in Drosophila larval classical olfactory conditioning.. PLoS One.

[64] Seugnet L, Suzuki Y, Vine L, Gottschalk L, Shaw PJ (2008). D1 receptor activation in the mushroom bodies rescues sleep-loss-induced learning impairments in Drosophila.. Curr Biol.

[65] Cassenaer S, Laurent G (2007). Hebbian STDP in mushroom bodies facilitates the synchronous flow of olfactory information in locusts.. Nature.

[66] Cassenaer S, Laurent G (2012). Conditional modulation of spike-timing-dependent plasticity for olfactory learning.. Nature.

